# A Comparison of Magnetic Resonance Imaging and Neuropsychological Examination in the Diagnostic Distinction of Alzheimer's Disease and Behavioral Variant Frontotemporal Dementia

**DOI:** 10.3389/fnagi.2016.00119

**Published:** 2016-06-16

**Authors:** Jingjing Wang, Stephen J. Redmond, Maxime Bertoux, John R. Hodges, Michael Hornberger

**Affiliations:** ^1^Graduate School of Biomedical Engineering, University of New South WalesSydney, NSW, Australia; ^2^Norwich Medical School, University of East AngliaNorwich, UK; ^3^School of Medical Sciences, University of New South WalesSydney, NSW, Australia

**Keywords:** machine learning, AD, bvFTD, classification, Bayesian, MRI

## Abstract

The clinical distinction between Alzheimer's disease (AD) and behavioral variant frontotemporal dementia (bvFTD) remains challenging and largely dependent on the experience of the clinician. This study investigates whether objective machine learning algorithms using supportive neuroimaging and neuropsychological clinical features can aid the distinction between both diseases. Retrospective neuroimaging and neuropsychological data of 166 participants (54 AD; 55 bvFTD; 57 healthy controls) was analyzed via a Naïve Bayes classification model. A subgroup of patients (*n* = 22) had pathologically-confirmed diagnoses. Results show that a combination of gray matter atrophy and neuropsychological features allowed a correct classification of 61.47% of cases at clinical presentation. More importantly, there was a clear dissociation between imaging and neuropsychological features, with the latter having the greater diagnostic accuracy (respectively 51.38 vs. 62.39%). These findings indicate that, at presentation, machine learning classification of bvFTD and AD is mostly based on cognitive and not imaging features. This clearly highlights the urgent need to develop better biomarkers for both diseases, but also emphasizes the value of machine learning in determining the predictive diagnostic features in neurodegeneration.

## Introduction

Clinical diagnosis of neurodegenerative diseases at clinical presentation remains challenging, in particular for phenotypologically similar diseases such Alzheimer's disease (AD) and behavioral variant frontotemporal dementia (bvFTD). Diagnostic criteria have been established and revised (Dubois et al., 2007; Rascovsky et al., 2011) for both diseases, with amnesia seen as a classic symptom of AD, whereas behavioral changes and executive impairments are reported as core criteria for bvFTD. However, recent evidence has highlighted that AD patients can present with dysexecutive and behavioral changes (Possin et al., 2013). Similarly, an important proportion of bvFTD patients, including pathologically confirmed patients, have been reported to show similar levels of amnesia as found in AD (Hornberger et al., 2010; Hornberger and Piguet, 2012; Bertoux et al., 2014).

These findings increase the challenge for clinicians in distinguishing between these two diseases at first presentation. One potential aid to the clinical diagnosis would be the use of machine/statistical learning algorithms to objectively interpret supportive diagnostic criteria (e.g., neuroimaging, cognition, etc.) to aid diagnosis based on the core diagnostic features. Such classifiers have been recently shown to accurately distinguish AD patients from healthy controls (Zhang et al., 2011; Zhou et al., 2014). However, classification against healthy individuals has limited utility as the distinction of neurodegenerative and healthy individuals is quite straightforward. More interesting would be to employ machine learning algorithms for the diagnostic distinction of different neurodegenerative diseases.

The current study addresses this issue by employing a Naïve Bayes classifier model to distinguish between a large clinical sample of individuals with clinically-diagnosed AD or bvFTD, as well as automatically separating these two disease classes from healthy age-matched controls at clinical presentation. Critically, a subset of patients had confirmed pathological diagnoses. Finally, to avoid circularity, we did not employ in the algorithm any core diagnostic features for the distinction of patients (such as the Cambridge Behavioural Inventory), as these features were used in the initial clinical diagnosis and provided the diagnostic reference against which the performance of the algorithm is compared (except for the pathologically-confirmed cases where pathology provided the final diagnosis); instead the algorithm utilizes diagnostic supportive features (i.e., atrophy neuroimaging and neuropsychology) only. Thus, our findings illustrate for the first time how supportive information can aid clinical diagnosis of these diagnostically challenging similar neurodegenerative conditions.

## Methods

### Participants

A total of 166 participants were selected (54 AD; 55 bvFTD; 57 healthy controls) from the FRONTIER (Frontotemporal Dementia Research Group) patient database, Sydney, Australia. All bvFTD patients met current consensus criteria (Rascovsky et al., 2011) with insidious onset, decline in social behavior and personal conduct, emotional blunting, and loss of insight. Patients with a known genetic mutation associated with bvFTD were not included in the study. All AD patients met revised NINCDS-ADRDA diagnostic criteria for probable AD (Dubois et al., 2007). Pathological confirmation of diagnosis was available for 22 patients (9 AD; 13 bvFTD).

Healthy controls were selected from a healthy volunteer panel or were spouses/carers of patients. The South Eastern Sydney and Illawarra Area Health Service and the University of New South Wales human ethics committees approved the study. Written informed consent was obtained from the participant or the primary caregiver in accordance with the Declaration of Helsinki.

### Neuropsychological assessment

All participants underwent cognitive screening using the Addenbrooke's Cognitive Examination (ACE-R; Mioshi et al., 2006). The ACE-R results in a score out of 100, and includes subsections in attention, memory, language and visuo-perception.

The frontotemporal dementia rating scale (FRS; Mioshi et al., 2010) was used to determine patients' disease severity. The Cambridge Behavioural Inventory (CBI; Wedderburn et al., 2008) was used as a behavioral disturbance measure.

Patients also underwent a comprehensive cognitive assessment including the Hayling test (Burgess and Shallice, 1996) that assess inhibition/response suppression, the backward digit span evaluating working-memory, lexical letter fluency tasks assessing verbal initiation, the Trail Making test (Reitan, 1955) evaluating flexibility, the recall of the Rey Complex Figure (Rey, 1941) as well as the Doors and People test (Baddeley et al., 1995), two visual memory tests, the Rey Auditory Verbal Learning Test (RAVLT–Rey, 1964) to assess verbal memory and a facial emotion recognition test based on Ekman faces (Ekman and Friesen, 1975). The cognitive assessments therefore covered extensive cognitive domains: executive (Digit Span; Hayling; FAS letter fluency; Trails); memory (Rey Figure Recall; RAVLT recall and recognition; Doors and People) and emotion recognition (Ekman faces test). Total or subscores of each test were employed in the Bayesian classification analysis.

### MRI acquisition and analysis

All patients and controls underwent the same imaging protocol to obtain whole-brain T1-weighted images using a 3T Philips MRI scanner with standard quadrature head coil (8 channels). The 3D T1-weighted sequences were acquired as follows: coronal orientation, 161 mm^2^ in-plane resolution, slice thickness 1 mm, TR/TE = 5.8/2.6 ms. MRI analysis was conducted using a Voxel-based morphometry (VBM) pipeline on three dimensional T1-weighted scans, using the FSL-VBM toolbox in the FMRIB software library package (http://www.fmrib.ox.ac.uk/fsl/). The first step involved extracting the brain from all scans using the BET algorithm in the FSL toolbox, using a fractional intensity threshold of 0.22. Each scan was visually checked after brain extraction, both to ensure that no brain matter was excluded, and no non-brain matter was included (e.g., skull, optic nerve, dura mater; Smith et al., 2004).

A gray matter template, specific to this study, was then built by canvassing 20 scans from each group (total *n* = 60). An equal number of scans across groups was used to ensure equal representation, and thus avoid potential bias toward any single group's topography during registration. Template scans were then registered to the Montreal Neurological Institute Standard space (MNI 152) using non-linear b-spline representation of the registration warp field, resulting in study-specific gray matter template at 2 × 2 × 2 mm^3^ resolution in standard space (Rueckert et al., 1999; Andersson et al., 2007). Simultaneously, brain-extracted scans were also processed with the FMRIB's Automatic Segmentation Tool (FAST v4.0) to achieve tissue segmentation into cerebrospinal fluid (CSF), gray matter and white matter. Specifically, this was done via a hidden Markov random field model and an associated expectation-maximization algorithm (Zhang et al., 2001).

The FAST algorithm also corrected for spatial intensity variations, such as bias field or radio-frequency inhomogeneities in the scans, resulting in partial volume maps of the scans. The following step saw gray matter partial volume maps then non-linearly registered to the study-specific template via non-linear b-spline representation of the registration warp. These maps were then modulated by dividing by the Jacobian of the warp field, to correct for any contraction/enlargement caused by the non-linear component of the transformation (Good et al., 2002). After normalization and modulation, smoothing the gray matter maps occurred using an isotropic Gaussian kernel (standard deviation = 3 mm; full width half maximum = 8 mm).

Based on the known spread of pathology in bvFTD and AD (Seeley et al., 2008), we *a priori* selected a subset of normalized, smoothed brain regions for the Bayesian classification analysis. The brain region boundaries were established via the cortical and subcortical Harvard-Oxford probabilistic atlases. The selected regions were the: (1) amygdala; (2) hippocampus; (3) medial temporal lobe; (4) temporal pole; (5) dorsolateral prefrontal cortex (DLPFC); (6) ventromedial prefrontal cortex (VMPFC); (7) striatum, and; (8) insula. For the selected regions, gray matter intensities were extracted and multiplied by the mean of the values in the smoothed registered gray matter to give total volume for each region and participant. The volumes were then corrected for total intracranial volume, as well as age and gender.

There is of course the opportunity to segment the brain images into smaller sub-regions, for example, into their left and right hemisphere sub-regions, but given the limited data set available with which to learn a pattern recognition model, we risk over-learning during the training phase. Therefore, we conservatively limit the pool to only eight MRI volumetric features.

### Data preparation

Participants were divided into three classes based on their disease classification (two disease classes, and one control class) as shown in Table 1.

**Table 1:** **Three classes of data, which include two disease classes, Alzheimer's disease (AD) and behavioral variant frontotemporal dementia (bvFTD), and a control group**.

	**AD (*n* = 54)**	**bvFTD (*n* = 55)**	**Controls (*n* = 57)**	***p*-values**
Age (years)	63.7 (8.1)	61.2 (9.4)	67.3 (6.8)	0.001
Gender (M/F)	31/23	37/18	25/32	0.043
Education (years)	12.3 (3.7)	12.3 (3.3)	13.1 (2.8)	0.138
Disease duration (years)	3.3 (2.1)	4.7 (3.3)	–	0.041

*Age, years of education, and disease duration are tested for group differences using Kruskal-Wallis tests. Gender is tested for group differences using Chi-squared test. Only education is shown not to be different between groups at 5% level of significance*.

For each participant, a vector of up to 25 numerical features was available, including the 8 MRI volumetric features and 17 neuropsychological features. This data was arranged in two data matrices, denoted as *X*_*scan*_ and *X*_*cog*_, respectively. The matrix concatenation of all data was also denoted as *X*_*all*_ = (*X*_*scan*_, *X*_*cog*_). Each row represents one subject and each column represents one feature variable.

As a number of neuropsychological cognitive scores were unavailable for several subjects, it is expected that this led to an underestimation of the discriminating capacity of these cognitive assessments in differentiating AD and bvFTD. A summary of the extent of this missing data is provided in Supplementary Table 1.

In order to compare the performance of a multivariate classifier model in discriminating the two disease classes of AD and bvFTD (then in discriminating between the three classes of AD, bvFTD and controls in a second step) using different combinations of the available features as the input, the following analyses were performed.

### Naïve Bayes classification

The Naïve Bayes classification method is adopted in this study primarily for its ability to handle missing features, which occurs for some of the neuropsychological assessments (Liu et al., 2005; Shi and Liu, 2011). A Naïve Bayes classifier is a simple probabilistic classifier based on the application of Bayes' theorem (described mathematically below) with the assumption of probabilistic independence between every pair of features; in practice this is rarely true, as certain features can be correlated, but Naïve Bayes classifiers demonstrate remarkably robust performance on features which are not strictly independent (Zhang, 2004). Given a discrete class label Y and n features, x_1_ through x_n_, Bayes' theorem states the following relationship:

(1) $$\begin{array}{l} \text{P}\left(\text{Y}|{\text{x}}_{1},\ldots ,{\text{x}}_{\text{n}}\right)=\frac{\text{P}\left(\text{Y}\right)\text{P}\left({\text{x}}_{1},\ldots ,{\text{x}}_{\text{n}}|\text{Y}\right)}{\text{P}\left({\text{x}}_{1},\ldots ,{\text{x}}_{\text{n}}\right)} \end{array}$$

where *P*(*Y*|*x*_1_, …, *x*_*n*_) is the posterior probability of class Y being correct given the observed features in the vector X = (*x*_1_, …, *x*_*n*_). Using the naïve independence assumption that features are independent of each other,

(2) $$\begin{array}{l} \text{P}\left({\text{x}}_{\text{i}}|\text{Y},{\text{x}}_{1},\ldots ,{\text{x}}_{\text{i}-1},{\text{x}}_{\text{i}+1},\ldots ,{\text{x}}_{\text{n}}\right)=\text{P}\left({\text{x}}_{\text{i}}|\text{Y}\right) \end{array}$$

the relationship is simplified to:

(3) $$\begin{array}{l} \text{P}\left(\text{Y}|{\text{x}}_{1},\ldots ,{\text{x}}_{\text{n}}\right)=\frac{\text{P}\left(\text{Y}\right)\prod_{\text{i}=1}^{\text{n}}\text{P}\left({\text{x}}_{\text{i}}|\text{Y}\right)}{\text{P}\left({\text{x}}_{1},\ldots ,{\text{x}}_{\text{n}}\right)} \end{array}$$

(4) $$\begin{array}{l} \text{P}\left(\text{Y}|{\text{x}}_{1},\ldots ,{\text{x}}_{\text{n}}\right)\propto \text{P}\left(\text{Y}\right)\prod_{\text{i}=1}^{\text{n}}\text{P}\left({\text{x}}_{\text{i}}|\text{Y}\right) \end{array}$$

(5) $$\begin{array}{l} \hat{\text{Y}}=\text{arg}\underset{\text{Y}}{\max}\text{P}\left(\text{Y}\right)\prod_{\text{i}=1}^{\text{n}}\text{P}\left({\text{x}}_{\text{i}}|\text{Y}\right) \end{array}$$

That is, the estimated class label which is output as a decision from the classifier model, denoted as $\hat{\text{Y}}$, is that which maximizes the expression $\;\text{ P}\left(\text{Y}\right)\prod_{\text{i}=1}^{\text{n}}\text{P}\left({\text{x}}_{\text{i}}|\text{ Y}\right)$.

The Naïve Bayes classifier used two steps to classify data, using the MATLAB Statistics and Machine Learning Toolbox 2014b (Mathworks, Natick, MA, USA):

- ***Training step***: Using training data, the method estimates the parameters of the probability distributions of x_i_ for each Y, assuming that the x_i_ are conditionally independent; that is, for each disease class Y, and each feature variable x_i_, the probability density P(x_i_|Y) is approximated with the available training data. In lay terms, P(x_i_|Y) is the probability of observing a value for the variable x_i_ given a particular disease class. The feature *x*_*i*_ can be either discrete or continuous, and either would suggest a different model for the probability density function, P(x_i_|Y). Since distributions are assumed independent, during training, missing instances for a particular feature are not included in the frequency count (for discrete variables) or distribution estimate (for continuous variables, using a Gaussian smoothing kernel function).
- ***Prediction step:*** For any unseen testing data, the method uses the previously estimated distributions to compute the value $\;\text{ P}\left(\text{Y}\right)\prod_{\text{i}=1}^{\text{n}}\text{P}\left({\text{x}}_{\text{i}}|\text{ Y}\right)$, which is proportional to the posterior probability, P(Y|x_1_, …, x_n_) (as shown above), for each possible class Y; either Y∈{*AD, bvFTD*} in the first analysis or Y∈{*AD, bvFTD, control*} in the second. The classifier then chooses the winning class, $\hat{\text{Y}}$, as the disease class which maximizes $\;\text{ P}\left(\text{Y}\right)\prod_{\text{i}=1}^{\text{n}}\text{P}\left({\text{x}}_{\text{i}}|\text{ Y}\right)$. During testing, for observations that have some but not all missing features, the algorithm estimates the class label using only non-missing features.

### 10-fold cross-validation

Rather than dividing the data evenly into training and testing sets, 10-fold cross-validation was used to obtain a better estimate of how the model will behave on a general data set by averaging out variations which were introduced by selecting one training/testing split from the data. The 109 AD and bvFTD subjects (or 166 subjects when also including controls) were randomly divided into 10 similar sized groups such that the proportion of subjects from each disease class was approximately equal within each group. For each of the 10 cross-validation runs, nine groups were used for training and the remaining group withheld for testing; this was repeated 10 times, such that each of the 10 groups were used as testing data for one of the 10 repeats. For any of the 10 repeats, given the training data from the other nine groups, the procedure for training the classifier is outlined above; however, it may be possible that the removal of some exceptionally noisy or highly correlated features before training may have improved the performance during the testing phase, therefore the following feature selection procedure was performed as a pre-processing step during the training phase of the classifier and not using any of the testing data for that repeat/fold.

### Feature selection

**Figure 1:**
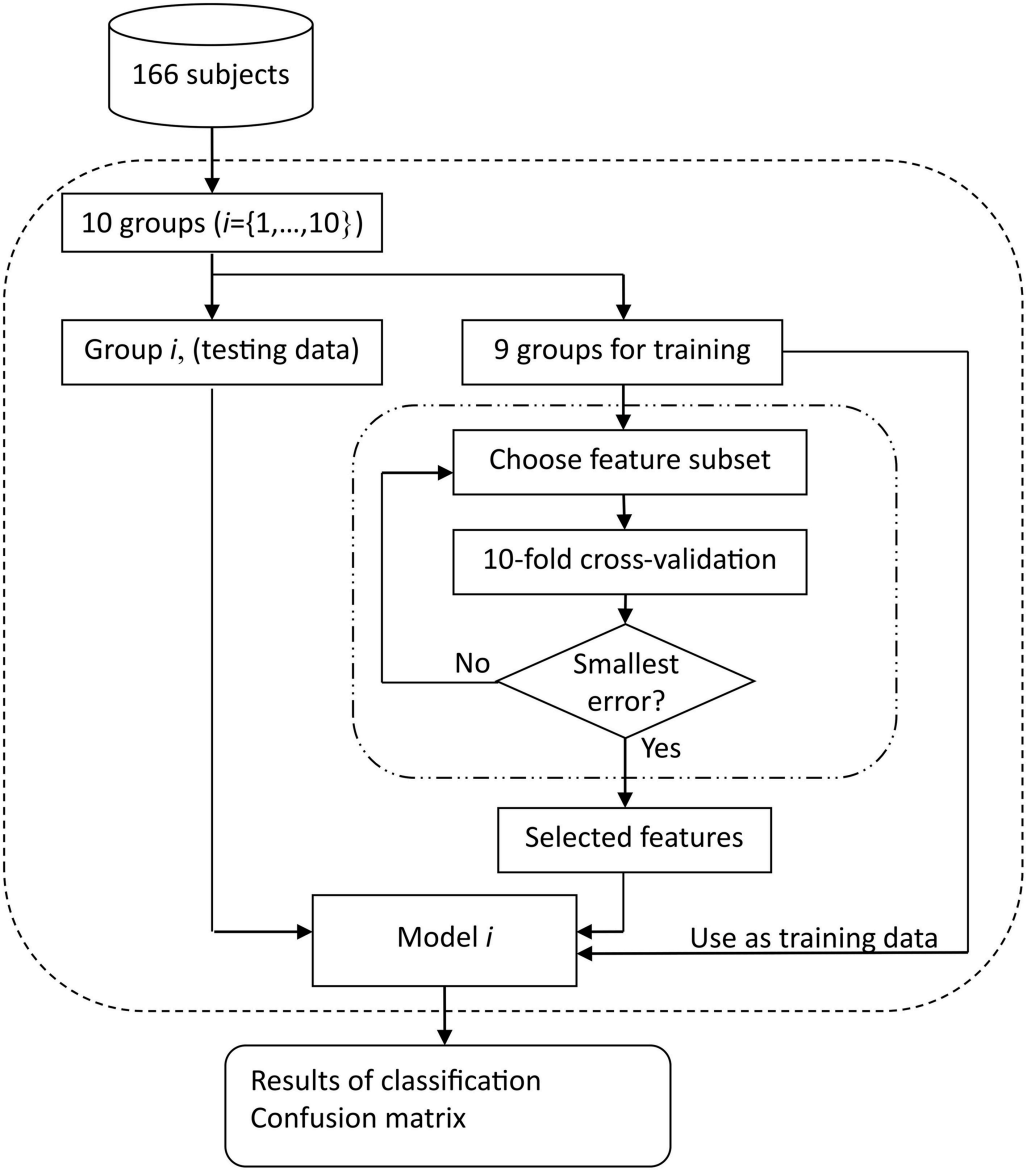
**Block diagram of training and testing of Naïve Bayes classification model**. One outer loop performs the testing, using 10 different groups with approximately 16 or 17 subjects in each group when *n* = 166 for three-way classification of AD, bvFTD, and control. The nine groups used for training in each run are subject to further feature selection to remove redundant or noisy features; each candidate feature subset is evaluated using an inner 10-fold cross-validation procedure.

As mentioned above, each training set contained data from nine subject groups. Starting with an empty candidate feature subset, features were sequentially added to the candidate subset until the addition of further features did not further improve the classification accuracy; this accuracy was determined using a second 10-fold cross-validation procedure within this training set in order to evaluate the potential feature subset under consideration. Figure 1 illustrates the entire process of classification and feature selection.

### Performance metrics

Classification performance was evaluated using both classification accuracy and Cohen's kappa statistic (Cohen, 1968). Approximate confidence intervals for accuracy were also listed; they were derived using the accuracy as calculated from the confusion matrix (pooling classification results from all 10 cross-validation repeats) and the number of subjects for which a classification result is obtained, so independence between classification results was not strictly observed (due to test data also being used as training data for other folds) as required when estimating confidence intervals. Confidence intervals were computed with the approximation that all results were drawn from a fixed classifier model (rather than cross-validation, which is actually used).

### Evaluating three different feature sets

In order to compare the usefulness of the MRI scans volumes and the neuropsychological assessment (cognitive and neuropsychiatric) features three different starting feature sets (before feature selection begins), *X*_*scan*_, *X*_*cog*_, and *X*_*all*_ were evaluated using the procedure shown in Figure 1.

## Results

### Classifying AD and bvFTD

Table 2 shows the classification results in discriminating AD and bvFTD (without considering the control group). Using the MRI volume features as input, the machine learning algorithm classified 51.4% (50% when considering only 22 confirmed cases) of bvFTD and AD patients correctly at presentation. In contrast, the neuropsychological scores achieved higher discrimination accuracy, correctly identifying 62.4% of bvFTD and AD cases. Not surprisingly, due to the low classification accuracy when using MRI volumes, the combined feature set (MRI volumes and neuropsychological) was only slightly decreased to 61.5% of correct discrimination between bvFTD and AD.

**Table 2:** **Results for classification of AD vs. bvFTD (*n* = 109)**.

		**Starting feature subset before feature selection**		
		**MRI volumes (8 features)**	**Neuropsychological/Neuropsychiatric (17 features)**	**All (25 features)**
Performance metric	Confusion matrix (22 confirmed cases)	$\begin{array}{cc} 36 & 35\\ 18 & 20 \end{array}\left(\begin{array}{cc} 8 & 10\\ 1 & 3 \end{array}\right)$	$\begin{array}{cc} 34 & 21\\ 20 & 34 \end{array}\left(\begin{array}{cc} 3 & 4\\ 6 & 9 \end{array}\right)$	$\begin{array}{cc} 32 & 20\\ 22 & 35 \end{array}\left(\begin{array}{cc} 4 & 6\\ 5 & 7 \end{array}\right)$
	Confusion matrix mean ± SD	3.6 ± 1.17 3.5 ± 1.27 1.8 ± 1.03 2.0 ± 0.94	3.4 ± 1.08 2.1 ± 1.10 2.0 ± 1.49 3.4 ± 1.07	3.2 ± 0.92 2.0 ± 1.15 2.2 ± 1.14 3.5 ± 1.18
	Cohen's kappa (Cohen's kappa for 22 confirmed cases)	0.03 (0.10)	0.25 (0.03)	0.23 (−0.02)
	Accuracy, 95% CI	51.38%, CI = [42.00%, 60.76%]	62.39%, CI = [53.30%, 71.48%]	61.47%, CI = [52.33%, 70.61%]
	(Accuracy, 95% CI for 22 confirmed cases)	(50.00%, CI = [29.11%, 70.89%])	(54.55%, CI = [33.74%, 75.36%])	(50.00%, CI = [29.11%, 70.89%])

*Each column of a confusion matrix represents the true class label, while each row represents the estimated class label. Within confusion matrices, the first columns/rows represent AD, while the second columns/rows represent bvFTD. The mean and standard deviation (SD) of each confusion matrix entry across the 10 cross-validation runs are also presented. Cohen's kappa coefficient and accuracy are calculated for the confusion matrix. The corresponding confirmed diagnoses are shown in parentheses. Approximate 95% confidence intervals (CI) are provided for classification accuracies*.

**Figure 2:**
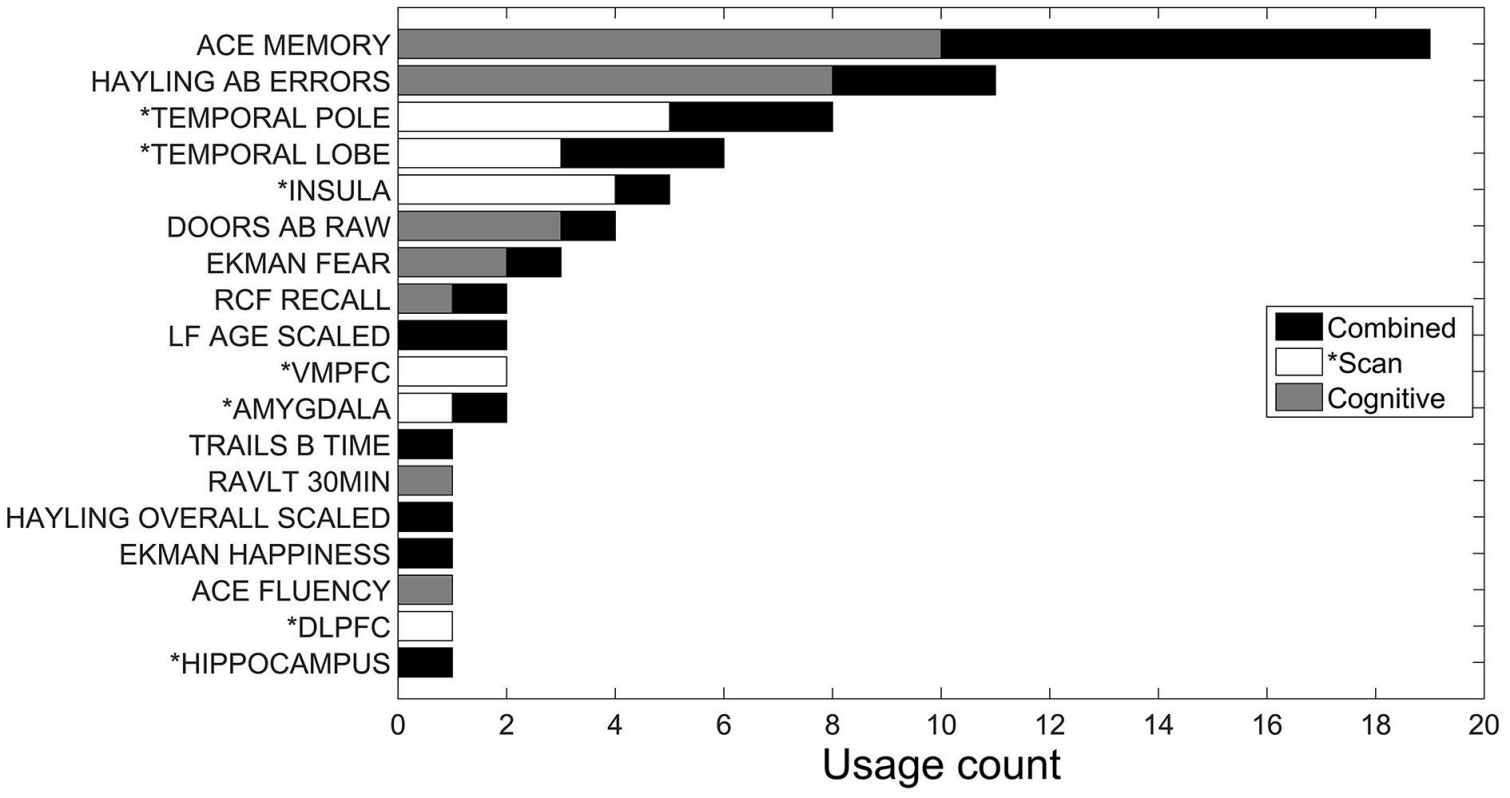
Accumulated feature selection results of 10-fold cross validation in discriminating AD and bvFTD using three different feature sets: MRI volumes (*Scan), neuropsychological (Cognitive), and both combined. Y-axis shows the name of selected features and X-axis shows the accumulated count of a corresponding feature being selected over the 10-folds. Three sets of features are displayed in different colors.

Figure 2 shows a histogram of the 10 sets of features selected for each of the 10 outer cross-validation runs, for a given starting feature set (derived from either the MRI volumes, neuropsychological assessment, or both combined). The higher the frequency with which the feature is selected, the more consistently it contributes to the classification task. There was a large variability across features contributing to successful discrimination. Using only MRI scan volume features (shown as white bars in Figure 2), six of the eight MRI regions were selected at least once, except for the striatum (which is never selected when discriminating between AD and bvFTD, and so not shown in Figure 2) and the hippocampus. The most selected regions were the temporal pole, insula, and temporal lobe. For the neuropsychological features (shown as gray bars in Figure 2), 7 of the 17 were selected at least once, with ACE-R memory subtest, Hayling AB errors, Doors and People test, and facial emotion recognition of fear scores being selected more than twice, and with the ACE-R memory subscore and Hayling AB errors being selected more than twice as often as the next most frequently selected neuropsychological feature (Doors and People test scores).

### Classifying AD, bvFTD, and controls

Table 3 shows the classification results in discriminating AD, bvFTD and control classes. MRI features achieved an accuracy of 54.2% (18.2%, when considering the 22 confirmed cases only). As in the previous classification, the three-class classification performed better using neuropsychological features, with an accuracy of 68.1%. The combination of both MRI and neuropsychological features achieves an accuracy of 67.5% (although confidence intervals overlap almost entirely).

**Table 3:** **Results for classification of AD, bvFTD, and control (*n* = 166)**.

		**Starting feature subset before feature selection**		
		**MRI volumes (8 features)**	**Neuropsychological/Neuropsychiatric (17 features)**	**All (25 features)**
Performance metric	Confusion matrix (confirmed cases)	$\begin{array}{ccc} 22 & 26 & 8\\ 14 & 19 & 0\\ 18 & 10 & 49 \end{array}\left(\begin{array}{ccc} 2 & 9 & 0\\ 1 & 2 & 0\\ 6 & 2 & 0 \end{array}\right)$	$\begin{array}{ccc} 29 & 15 & 0\\ 22 & 31 & 4\\ 3 & 9 & 53 \end{array}\left(\begin{array}{ccc} 3 & 4 & 0\\ 5 & 6 & 0\\ 1 & 3 & 0 \end{array}\right)$	$\begin{array}{ccc} 29 & 17 & 0\\ 19 & 28 & 2\\ 6 & 10 & 55 \end{array}\left(\begin{array}{ccc} 5 & 5 & 0\\ 2 & 5 & 0\\ 2 & 3 & 0 \end{array}\right)$
	Confusion matrix mean ± SD	2.2 ± 1.23 2.6 ± 1.26 0.8 ± 0.63 1.4 ± 1.26 1.9 ± 1.29 0.0 ± 0.00 1.8 ± 1.14 1.0 ± 0.82 4.9 ± 0.88	2.9 ± 1.37 1.5 ± 1.08 0.0 ± 0.00 2.2 ± 1.75 3.1 ± 1.20 0.4 ± 0.70 0.3 ± 0.95 0.9 ± 0.88 5.3 ± 0.82	2.9 ± 0.99 1.7 ± 1.16 0.0 ± 0.00 1.9 ± 1.29 2.8 ± 0.92 0.2 ± 0.63 0.6 ± 0.97 1.0 ± 1.05 5.5 ± 0.71
	Cohen's kappa (Cohen's kappa for confirmed cases)	0.31 (−0.14)	0.52 (−0.03)	0.51 (0.13)
	Accuracy, 95% CI	54.22%, CI = [46.64%, 61.80%]	68.07%, CI = [60.98%, 75.16%]	67.47%, CI = [60.34%, 74.60%]
	(Accuracy, 95% CI for 22 confirmed cases)	(18.18%, CI = [2.06%, 34.30%])	(40.91%, CI = [20.36%, 61.46%])	(45.45%, CI = [24.64%, 66.26%])

*Each column of a confusion matrix contains the actual disease diagnosis, while the rows contain the disease class estimated by the classifier. The first, second, and third columns/rows represent AD, bvFTD, and control, respectively. Corresponding results for confirmed diagnoses are shown in parentheses. Approximate 95% confidence intervals (CI) are provided for classification accuracies*.

**Figure 3:**
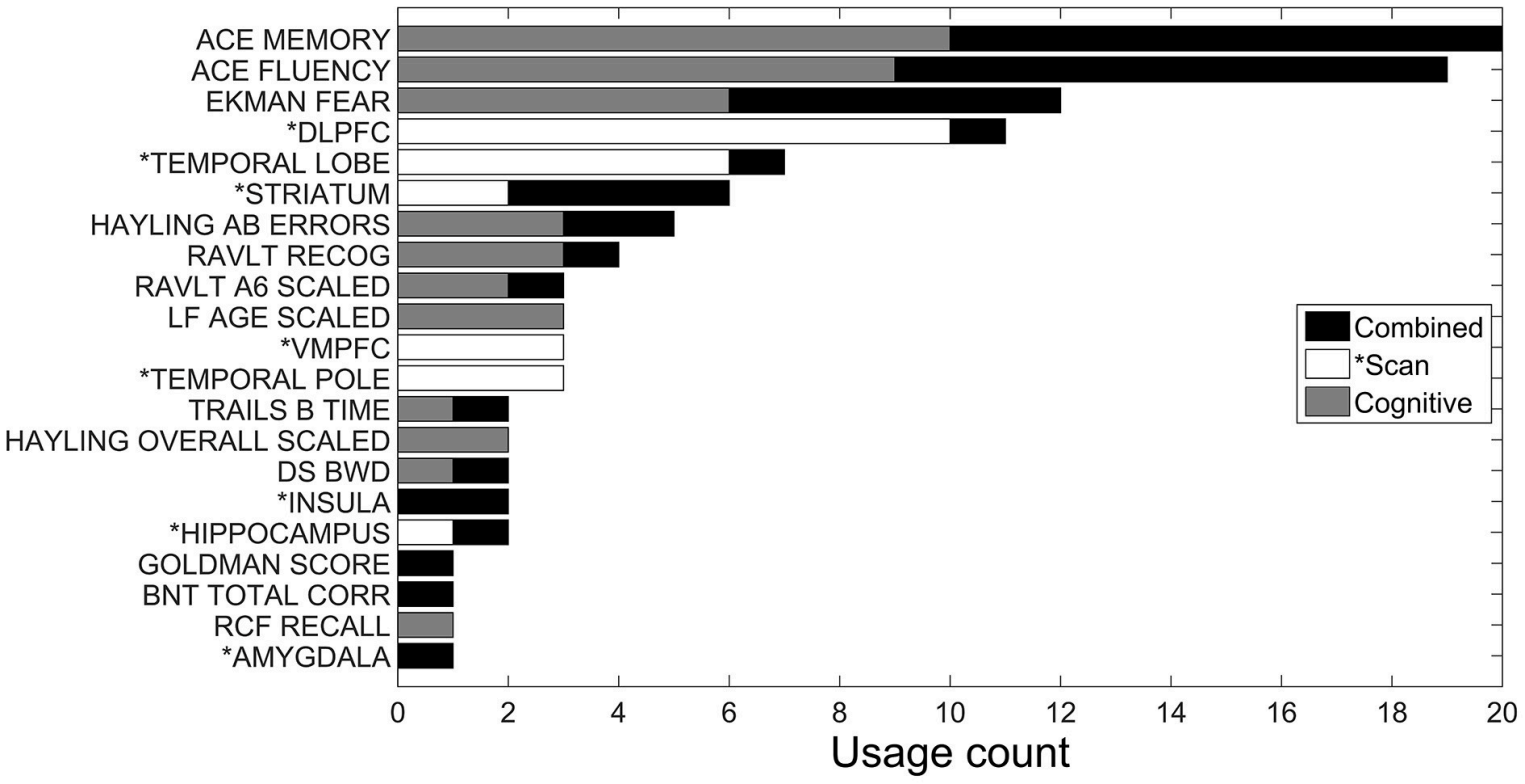
Accumulated feature selection results of 10-fold cross validation in discriminating AD, bvFTD, and control classes using three different feature sets: MRI volumes (*Scan), neuropsychological (Cognitive), and both combined. Y-axis shows the name of selected features and X-axis shows the accumulated count of a corresponding feature being selected over the 10-folds. Three sets of features are displayed in different colors.

The corresponding feature selection results are shown in Figure 3. The most selected features when using only MRI features were the DLPFC, temporal lobe, VMPFC and temporal pole. When using neuropsychological features, the most commonly selected features were ACE-R memory and ACE-R fluency subscores as well as facial emotion recognition of fear. Combining all (neuropsychological and imaging) features in the analysis, these same three neuropsychological features remained among the most selected, however, DLPFC and temporal lobe (which were the two most frequently selected features when using only MRI scan features) are each only selected for one of the 10 cross-validation runs. This last result indicates that the neuropsychological features already contained this same scan information. Interestingly, when combining both scan and neuropsychological features, the striatum is selected twice as often (rising from being selected twice to being selected four times).

## Discussion

To our knowledge, this is the first study investigating the use of machine learning algorithms to differentiate AD and specifically bvFTD. Results showed that neuropsychological scores and particularly tests of emotion recognition, memory screening and executive assessment achieved the best classification results. Cortical volumes of a subset of frontal, temporal, and insular regions were the most distinctive anatomical features to distinguish the groups.

Previous neurodegenerative machine learning studies have virtually been all focused on AD and its prodromal stages (Walhovd et al., 2010; Cuingnet et al., 2011; Hinrichs et al., 2011; Zhang et al., 2011; Zhou et al., 2014), whereas only one study examined discriminating AD from more general frontotemporal lobar degeneration (FTLD; Klöppel et al., 2008) as a clinical spectrum. In addition, virtually all these studies have focused mostly on neuroimaging features, and none have attempted to distinguish between the specific diseases of AD and bvFTD, whereas the current study used additional neuropsychological features as well as a pathologically confirmed bvFTD patient subgroup.

On a cognitive level, the most salient neuropsychological features to accurately classify AD and bvFTD were assessment of emotion recognition (Ekman faces), inhibition (Hayling), visual episodic memory (Doors and People), and verbal memory screening (ACE-R memory). These findings nicely corroborate previous results showing that, at presentation, emotion recognition deficits and disinhibition are hallmarks of bvFTD while being relatively absent in AD (Hornberger et al., 2011; Bertoux et al., 2015). In contrast, AD patients' prevalent episodic memory problems were most distinctive for this patient group, although some bvFTD can show impaired episodic memory performance (Hornberger et al., 2010; Bertoux et al., 2014). More specifically, a subgroup of bvFTD patients can show severe episodic memory problems, which limits the utility of episodic memory problems in the diagnostic distinction of both diseases. Future machine learning approaches on such amnestic bvFTD compared to AD patients would be of importance to confirm this notion. Finally, similar neuropsychological factors were found to discriminate groups when controls were also added in the analysis, further corroborating the robustness of the findings.

On an anatomical level, the temporal pole and insula were the most distinctive features to distinguish between AD and bvFTD. The insula has been previously shown to be among the earliest of the regions atrophic in bvFTD (Perry et al., 2006) and is selectively impaired compared to AD. The identification of the temporal lobe as a significant feature to distinguish both diseases is an intriguing result, as both AD and bvFTD show significant changes in this region. Nevertheless, the atrophy of the temporal pole, which accounts for a large part of the temporal lobe, might explain this finding, as it is indeed strongly associated with bvFTD pathology (Whitwell et al., 2009). The atrophy findings are therefore strongly dominated by the bvFTD atrophy pattern spanning temporal pole and insular regions, whereas interestingly prefrontal cortex regions (DLPFC, VMPFC) as well as medial temporal lobe regions contributed little to the classification accuracy. This is further confirmed by the analysis including the controls, which only then showed volumes of the VMPFC and DLPFC as well as of the temporal lobe and pole strongly contributing to the classification.

Interestingly, neuropsychological features outperformed cortical volume features for the classification accuracy between bvFTD and AD (62.4 vs. 51.4%, for cortical volume or neurophysiological features, respectively). More intriguing is the fact that the combination of atrophy and neuropsychological features did not increase the classification accuracy. This indicates a redundancy in the variables with neuroimaging and cognitive features seemingly representing the same dysfunction. Finally, similar classification results were observed when the analysis was restricted to the pathologically confirmed cases for which the neuropsychological measures showed a classification rate of 54.6% and atrophy features an even a lower accuracy rate of 50.0%. It is likely that the difference in sample size between the overall group (*n* = 109) and the pathological confirmed cases (*n* = 22) may explain the difference of classification accuracy for the combining features between the analyses (62.4% for *n* = 109, and 54.6% for *n* = 22). Still, it is important to note that classification results were relatively similar in the pathological subgroup as it still represents the gold standard of definite diagnosis in both diseases.

It is interesting to note that the previous study by Klöppel et al. (2008) achieved much higher sensitivity and specificity (94.7 and 83.3%, respectively) using MRI atrophy contrasts of AD and FTLD, showing that parietal and frontal changes were particularly informative in the distinction of AD and FTLD, respectively. However, the inclusion of language-variant FTLD together with behavioral-variant, as well as the exclusion of bvFTD patients with memory impairment could explain the difference with our results, as it has been shown that AD and bvFTD can overlap to a large degree for scan-based measures (Hornberger and Piguet, 2012; Hornberger et al., 2012; De Souza et al., 2013), whereas other FTLD clinical subtypes (sv-FTD; nfv-PPA) show more distinct scan features (Gorno-Tempini et al., 2011). Also, a key differences between Klöppel et al.'s study and ours is that we used more specific regions (e.g., VMPFC) as neuroimaging features instead of the entire cortical lobes (e.g., frontal lobe), which may have lowered the general discriminative power.

Another novelty in our study was the employment of a three-way classification (AD, bvFTD, and controls) in a *post-hoc* analysis, which allowed contrasting the patient groups with controls at the same time. While it is not possible to directly compare these results with other reports in the literature, an approximate comparison can be made against several reported attempts to distinguish AD from controls. Previous studies showed good sensitivity/specificity (>80% sensitivity and >90% specificity) of imaging measures to distinguish AD from controls (Hamelin et al., 2015). In our results (Table 3), using the neuroimaging features resulted in 8 normal controls being erroneously classified as AD patients, and 28 diseased patients (18 AD and 10 bvFTD) wrongly classified as normal. In contrast, using neuropsychological scores instead in the model resulted in much fewer errors when classifying between controls and patients. Interestingly, these results are similar to Hinrichs et al. (2011) which reported that both cognitive and neuroimaging features contributed to the prediction of MCI patients progressing to full-blown AD—with neuroimaging features contributing slightly more to the classification. As mentioned already above, it is currently not clear how much cognitive and neuroimaging atrophy features map onto each other, however, it becomes apparent that even if there is some redundancy, a complementary diagnostic and classification approach can potentially corroborate diagnosis based on only one feature. There is clearly great scope to explore this further in the future, in particular in the distinction of neurodegenerative conditions from each other.

Despite these promising results there are limitations to our findings. In particular, only a subset of patients had a pathologically confirmed diagnosis. Ideally, we would have pathological confirmation in all patients. Still, the pathological confirmed participants showed similar results to the clinical cohort. A further limitation might have been the selection of specific neuroimaging and cognitive features in the analysis. As outlined in the methods, the *a priori* reasoning was to include features that have been shown to be most sensitive and specific to the respective pathologies. However, this might mean that other features which potentially could have allowed better classification were not considered in the current analysis. There may also be a small positive bias in the results due to the registration of brain images prior to the machine-learning exercise performed herein (that is, images are normalized using available data outside of the cross-validation loop); however, failing to perform such registration would likely lead to a larger negative bias in results due to the effects of age and gender covariates which also correlate with tissue volumes. Missing data among the neuropsychological assessment features will also have resulted in a lesser reported accuracy than what is achievable if these data were complete; hence, neuropsychological assessment could outperform MRI scans in this diagnostic task by a greater margin than what is presented herein. Finally, despite the sample size being excellent for clinical studies, the current sample size poses a challenge for modeling techniques, such as the one used here. In particular, the sample size relative to number of features can lead to worse performance than true performance in wild due to overfitting during feature selection and training; i.e., large variation in features selected between cross-validation runs. It would be therefore important to replicate our results in independent and larger samples in the future. Still, we believe that the current findings are of importance and highlight how, in the near future, clinicians could use novel computational techniques at a single patient level to aid their clinical diagnoses.

Taken together, this study used a machine learning classifier to distinguish AD and bvFTD. Despite showing promising findings, the separability of the three groups, and in particular between the two patient groups, was lower than expected. Cortical volume in temporo-insular regions allowed a classification accuracy of 51.4% between AD and bvFTD, while neuropsychological scores of emotion recognition, cognitive inhibition and memory reached approximately 62.4% accuracy. These results suggest that machine-learning classifier for AD and bvFTD should rely more on cognitive performance than cortical volumes and can provide clinicians with objective supportive information under diagnostic uncertainty.

## Author contributions

JW and SR analyzed the data and developed the model. MB, JH, and MH did the experiments and obtained data from patients. All co-authors have contributed writing the manuscript as well as proof-reading.

### Conflict of interest statement

The authors declare that the research was conducted in the absence of any commercial or financial relationships that could be construed as a potential conflict of interest.
