# Supplementary material for: A Comparison of Magnetic Resonance Imaging and Neuropsychological Examination in the Diagnostic Distinction of Alzheimer's Disease and Behavioral Variant Frontotemporal Dementia
Source: Front Aging Neurosci. 2016 Jun 16;8:119. doi: 10.3389/fnagi.2016.00119 (PMC4909756; doi:10.3389/fnagi.2016.00119)
Supplement: Supplementary file 1 [file Table1.DOCX]

Supplementary Material

Utility of Machine Learning Algorithms in the Diagnostic Distinction of Alzheimer’s Disease and Behavioral Variant

Frontotemporal Dementia

J. Wang^1^, S. J. Redmond^1^, M. Bertoux ^2^, J. R. Hodges^3^, M. Hornberger^2*^

^1^Graduate School of Biomedical Engineering, University of New South Wales, Sydney, Australia

^2^Norwich Medical School, University of East Anglia, Norwich, UK

^3^School of Medical Sciences, University of New South Wales, Sydney, Australia

*** Correspondence: Prof. Michael Hornberger,** Norwich Medical School, University of East Anglia, Norwich, NR4 7TJ, UK

[m.hornberger@uea.ac.uk](mailto:m.hornberger@uea.ac.uk)

# Supplementary Tables

**Supplementary Table 1. Missing feature count for neuropsychological/neuropsychiatric assessment**. *k* denotes the number of missing features for a given subject out of a total 17 such features. *n* denotes the number of subjects who were missing *k* assessment features. For example, 75 subjects had a complete set of cognitive assessment data. In total, 345 subject assessment features are missing from the feature matrix $X_{cog}$, which contains 2,822 (166×17) entries when considering all 166 subjects, or 1,853 (109×17) entries when considering the 109 AD and bvFTD subjects.

| *k* | 0 | 1 | 2 | 3 | 4 | 5 | 6 | 7 | 9 | 10 | 11 | 12 | 13 | 14 | 15 |
| --- | --- | --- | --- | --- | --- | --- | --- | --- | --- | --- | --- | --- | --- | --- | --- |
| *n* | 75 | 34 | 20 | 6 | 6 | 4 | 5 | 1 | 1 | 5 | 2 | 3 | 2 | 1 | 1 |
